# Supplementary figures and images for: Evolution Characterization and Pathogenicity of an NADC34-like PRRSV Isolated from Inner Mongolia, China
Source: Viruses. 2024 Apr 26;16(5):683. doi: 10.3390/v16050683 (PMC11125647; doi:10.3390/v16050683)

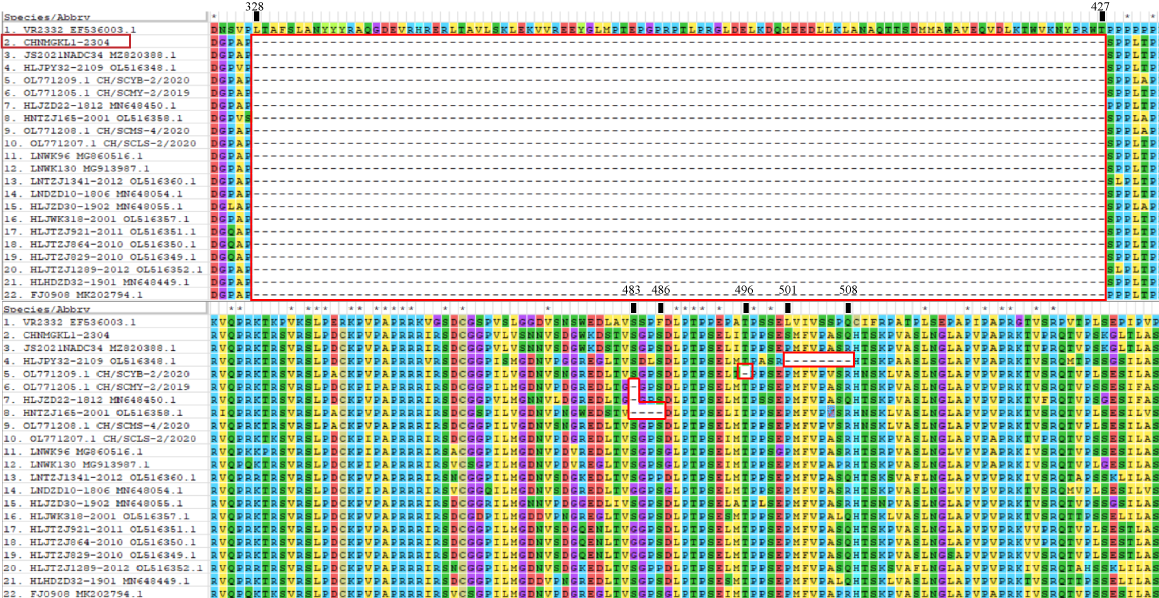

Supplement: Supplementary file 1 [file viruses-16-00683-s001.zip › Figure S2.tif]

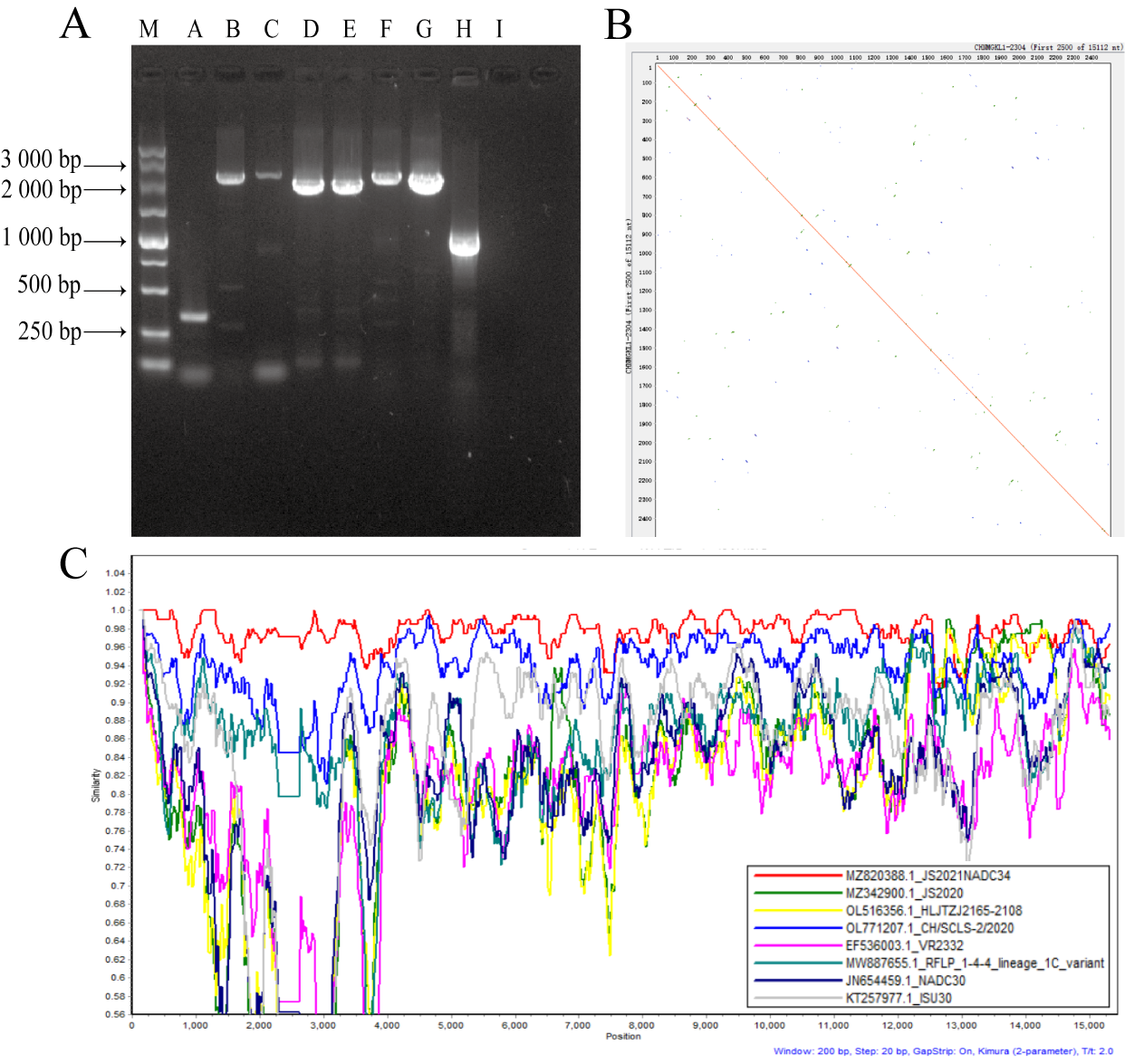

Supplement: Supplementary file 1 [file viruses-16-00683-s001.zip › Figure S1.tif]
